# Climate and other environmental factors predict tick abundance and Lyme cases in Minnesota one and two years in advance

**DOI:** 10.1016/j.onehlt.2026.101507

**Published:** 2026-07-03

**Authors:** Kathleen E. Angell, Janet Jarnefeld, Elizabeth K. Schiffman, M. Jana Broadhurst, Jianghu James Dong, Abraham Degarege, Roberto Cortinas, David M. Brett-Major

**Affiliations:** a Department of Epidemiology, College of Public Health, University of Nebraska Medical Center, Omaha, NE, United States of America; b Metropolitan Mosquito Control District, Saint Paul, MN, United States of America; c Minnesota Department of Health, St. Paul, MN, United States of America; d Global Center for Health Security, University of Nebraska Medical Center, Omaha, NE, United States of America; e Department of Pathology, Microbiology, and Immunology, College of Medicine, University of Nebraska Medical Center, Omaha, NE, United States of America; f Department of Biostatistics, College of Public Health, University of Nebraska Medical Center, Omaha, NE, United States of America; g School of Veterinary Medicine and Biomedical Sciences, University of Nebraska, Lincoln, NE, United States of America

**Keywords:** *Ixodes scapularis* tick, *Dermacentor variabilis* tick, Vector prevalence, Disease surveillance, Lyme disease, Midwest, Climate

## Abstract

**Background:**

Environmental factors, like weather and host abundance influence tick populations which in turn affect tickborne disease in endemic regions. It is important to understand how these factors are associated with tick abundance, and whether they can predict disease. We assessed associations between environmental factors, tick abundance, and human Lyme disease cases in the same year and with one- and two-year lags in Minnesota. In parallel, we compare conventional analytical methods with a novel machine learning approach to evaluate their relative strengths and potential for integration.

**Methods:**

Environmental and tick abundance relationships were examined using generalized linear mixed-effects models (GLMM), and gradient boosting machine learning incorporating same-year and time lagged effects.

**Results:**

Area under the curve (AUC) indicates higher accuracy in predicting tick abundance in GLMM than gradient boosting. Among GLMM with no time lag, Palmer Drought Severity Index (PDSI), vapor pressure deficit (VPD), precipitation and snow water equivalent (SWE) were significant predictors of tick abundance. Direction of association varies in PDSI and SWE variables with no time lag but show consistency with one- and two-year lags. Among gradient boosted models, days below −18 °C, small mammal count, and mouse-to-small mammal ratio were associated with high tick abundance in the same year, and with one- and two-year lags. AUC are highest with a one-year lag suggesting environmental factors most accurately predict tick abundance one year later. AUC in models with Lyme disease as the outcome are highest with a two-year lag; PDSI, soil moisture, SWE, days below −18 °C, degree days, small mammal count and mouse ratio are all variables of importance in gradient boosted models.

**Conclusion:**

Monitoring environmental factors provides enhanced opportunities for public health interventions through prediction of tick abundance and potential consequences for higher Lyme disease incidence. Incorporating traditional and modern analytic methods offers opportunities for enhanced prediction and early warning.

## Highlights

• Environmental factors provide early warning of high tick abundance in the following year
• Environmental factors can accurately predict increased risk for high Lyme cases two years in advance
• Machine learning augments traditional methods to predict tick abundance and Lyme disease incidence

## Graphical abstract

**Figure:**
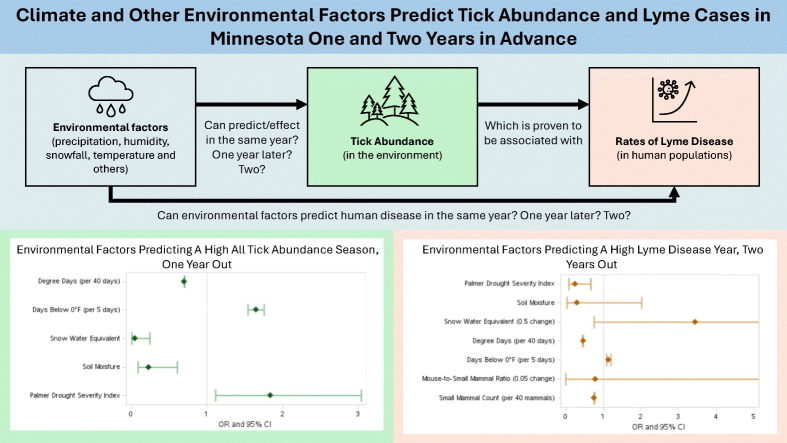

## 1 Background

Environmental factors such as physical environment, weather and host prevalence and diversity influence tick abundance, which in turn is associated with human risk for tick borne diseases [1], [2], [3], [4], [5]. Lyme disease, a nationally notifiable disease, is the most common vector borne disease reported in Minnesota and the United States [6], [7]. In the upper Midwest, the causative agents for Lyme disease, *Borrelia burgdorferi* sensu stricto and *Borrelia mayonii*, are present in *Ixodes scapularis* ticks. These ticks can also transmit the pathogens responsible for anaplasmosis, babesiosis, and ehrlichiosis. *Dermacentor variabilis*, another commonly encountered tick in the region, can spread Rocky Mountain spotted fever and tularemia, emerging diseases of concern in Minnesota [3], [6]. Given the threats these diseases pose, it is important to fully understand the associations between environmental factors and tick abundance.

Much of the available literature on environmental factors and their effect on tick abundance in the United States is focused on the Northeast region, leaving the Midwest underrepresented. Climatic variables like temperature and precipitation exhibit contrasting relationships with tick abundance between these two regions [8]. Additionally, *I. scapularis* biology varies geographically; Northeastern cohorts display asynchronous seasonal peaks, whereas the Upper Midwest experiences overlap between larval and nymphal life stages [9]. Further, regional genotypes influence tick lifespans, with Midwest populations demonstrating unique hardiness to local climatic stressors compared to their Northeastern counterparts [10]. A more localized, in-depth exploration is required to accurately model and understand these dynamics in Minnesota.

Most investigations into prediction of tick abundance have employed conventional analytic techniques in model building [2], [3], [8]. Gradient boosting machine learning trains models sequentially, combining weak learners into a strong learner model. Gradient boosting represents an opportunity to better understand how environmental factors affect tick abundance by incorporating variables that may impact ticks in tandem with other factors rather than in isolation and so would be discarded in traditional analytic techniques.

We brought together publicly available meteorologic data coupled with outputs of the Metropolitan Mosquito Control District's (MMCD) [7] active tick surveillance, and the Minnesota Department of Health's (MDH) active Lyme disease surveillance, creating a unique opportunity to explore environmental factor effects on tick abundance and disease transmission in the understudied Midwest region. Recognizing that tick populations are driven by both abiotic and biotic factors, we sought to examine how environmental variables and host availability influence tick abundance and assess whether these factors can predict human cases of disease. We also evaluated potential time-lag effects of environmental variables on both tick abundance and human disease. This accounts for delays inherent in tick life-cycle transitions, as well as clinical lag between exposure, symptom onset, and case reporting. These relationships were analyzed using both traditional analytic and machine learning methods, with particular emphasis on comparing their performance and exploring how integration of the two could improve predictive accuracy.

## 2 Methods

### 2.1 Data sources & sets

The data used in this study were obtained from MMCD (Tick Surveillance), TerraClimate (Climate Data), Minnesota Department of Natural Resources (MNDNR) (Snow Fall Data), and MDH (Disease Surveillance).

#### 2.1.1 Tick surveillance

MMCD began active vector surveillance in the seven-county Twin Cities metropolitan area in 1990. MMCD conducts annual vector surveillance through small mammal trapping from the last week of April to the last week of October across one hundred randomly selected sites using a square-mile sampling region within each township. Trapping and removal of on-host ticks is performed on a subset of sites, rotating weekly so that each location is measured three times within the twenty-seven-week sampling period. A detailed description of sites, trapping method, species identification, etc. has been previously described [3].

MMCD provided tick data from 2014 to 2023 in the form of a line list with each line representing one trapped small mammal. Host species and sex, number of ticks, week of surveillance period, date, and trapping location were included. The number of *D. variabilis* and *I. scapularis* ticks found on each animal was recorded and categorized by larva or nymph life stage. To explore differences in tick abundance measures at the county level, tick density was calculated as the total number of ticks divided by the total number of surveilled mammals within a given county and year; tick intensity was calculated as the total number of ticks divided only by the number of tick-infested mammals within that county and year. Density and intensity metrics were calculated both overall and stratified by tick species (*D. variabilis* and *I. scapularis*) at the county level. The total number of trapped small mammals was summed by county and year regardless of tick attachment. Further, the ratio of *Peromyscus leucopus* (white-footed mouse) to all other trapped mammals was calculated by dividing the number of *P. leucopus* by the total number of small mammals trapped in that county and year.

#### 2.1.2 Climate data

TerraClimate is a high-spatial-resolution (∼4 km) gridded global dataset of climate data from 1958 to present [11]. Daily climate data from 2014 to 2023 were extracted for the seven-county Twin Cities metropolitan region using TerraClimate's spatial aggregation tool, which computes area weighted averages over defines US county polygons. Minimum and maximum daily temperatures were used to calculate degree days and the number of cold snap days in a month. Degree-days were calculated by subtracting 11 from the mean daily temperature ((minimum daily temperature + maximum daily temperature) / 2); one degree day accumulated for each degree above the set threshold. The degree day threshold of 11 °C was chosen based on estimated thermal growing needs for larval *I. scapularis* emergence [12]. Degree day accumulation was calculated from January through May to get a sense of temperatures conducive to tick growth in late winter and spring leading into the tick season. Cold snap days were calculated as the accumulated number of days in the winter season (November to April) that dropped below −18 °C. The cold snap temperature threshold was chosen based on estimated temperatures needed for tick mortality of *I. scapularis* larva and nymphs [13]. Total precipitation in inches, total snow water equivalent (SWE) in inches, total column soil moisture in inches, average vapor pressure deficit (VPD) in kPa, and average Palmer Drought Severity Index (PDSI) were extracted by county and month. Mean precipitation, soil moisture, VPD and PDSI were calculated for the tick season (April through October). Mean SWE was calculated for the winter season (November through March).

#### 2.1.3 Snow fall data

MNDNR offers open-source historical climate data from several monitoring stations across the Twin-Cities metropolitan area. Daily snow depth measurements from 2013 to 2023 were downloaded by county. Mean snow depth in inches for the winter season was calculated setting days with trace snow to zero inches.

#### 2.1.4 Disease surveillance

MDH conducts surveillance of statewide human Lyme disease cases in accordance with nationally notifiable disease requirements [14]. The number of reported cases meeting surveillance classifications for probable or confirmed cases, by county, for the seven-county metropolitan area from 2014 to 2023 was provided. Prior to 2022, case counts include probable and confirmed cases of Lyme disease; since 2022, the numbers include probable cases only because of a change in the case definition removing the confirmed case classification for high incidence states like Minnesota. Probable and confirmed cases prior to 2022 were combined for comparability.

### 2.2 Statistical analyses

The following statistical analyses were conducted using SAS Version 9.4, with the exception of gradient boosting, which was performed in RStudio using the gbm package. An alpha level of 0.05 was used to determine statistical significance across all analyses. We performed descriptive analyses to assess annual trends and variation in tick abundance and cases of Lyme disease, summarizing environmental factors, stratifying the data by high and low tick years, which were defined as falling within the upper and lower 50th percentiles by county. To model the potential predictors of tick abundance, we utilized logistic regression to determine the odds of a year being high or low tick abundance based on predicting variables (PDSI, VPD, soil moisture, precipitation, SWE, snow depth, cold snap days, degree days, small mammal count and mouse ratio). Tick data was analyzed dichotomously as high or low tick abundance, density, and intensity for all ticks, and stratified by *I. scapularis*, and *D. variabilis*. After reviewing the continuous distribution of the predicting variables across the binary outcomes, we categorized VPD and mean precipitation rather than modeling them as continuous variables. VPD was dichotomized into high and low categories using a cutoff threshold of 0.85, while mean precipitation was categorized as low, normal and high using cutoff thresholds of 3.5 and 4.5, respectively.

#### 2.2.1 Predicting tick prevalence using backward selection

To explore the relationship between environmental factors and tick prevalence measures, we employed generalized linear mixed-effects models (GLMM), incorporating county as a repeated measure. All predicting variables were considered for inclusion in the model using backward selection (α = 0.05).

#### 2.2.2 Predicting tick prevalence using gradient boosting

To explore variables of importance and their relative influence on tick prevalence measures, gradient boosting was performed in RStudio with a Bernoulli distribution. Variable importance with relative influence was produced. Model accuracy by number of included variables was plotted to determine the optimal number of variables for maximum model accuracy.

To compare accuracy of the models based on the gradient boosting technique, output from gradient boosting was translated to GLMM adjusting for county as a repeated measure. Model accuracy by number of included variables was plotted to determine how many variables should be included in the GLMM. Utilizing the most influential predictors identified during the analysis, model performance was further assessed by calculating the Area Under the Curve (AUC) for both the backward selection process and the gradient boosting models to facilitate a direct comparison of predictive capabilities.

#### 2.2.3 Predicting lyme disease cases from environmental factors

To explore whether environmental factors could be further used to predict years with higher numbers of Lyme disease cases, GLMM was used with binary distribution adjusting for county as a repeated measure with cases of Lyme disease as the outcome (dichotomized as high or low based on the median). Backward selection using an alpha threshold of 0.05 was used to determine variable inclusion in the model. Gradient boosting was also performed in RStudio with the same methods as previously described.

#### 2.2.4 Time lag effects on predicting tick prevalence and lyme disease cases

To investigate potential time lag effects of environmental factors on both tick abundance and subsequent human disease, we re-evaluated the aforementioned models by incorporating one- and two-year lag terms for the environmental predictors. The resulting outputs were compared to assess which effects are likely to predict vector abundance and disease cases the same year, the following year, and two years following. This analysis provided insight into both immediate and delayed effects of environmental conditions on tick populations and Lyme disease cases in the human population.

## 3 Results

**Fig. 1:**
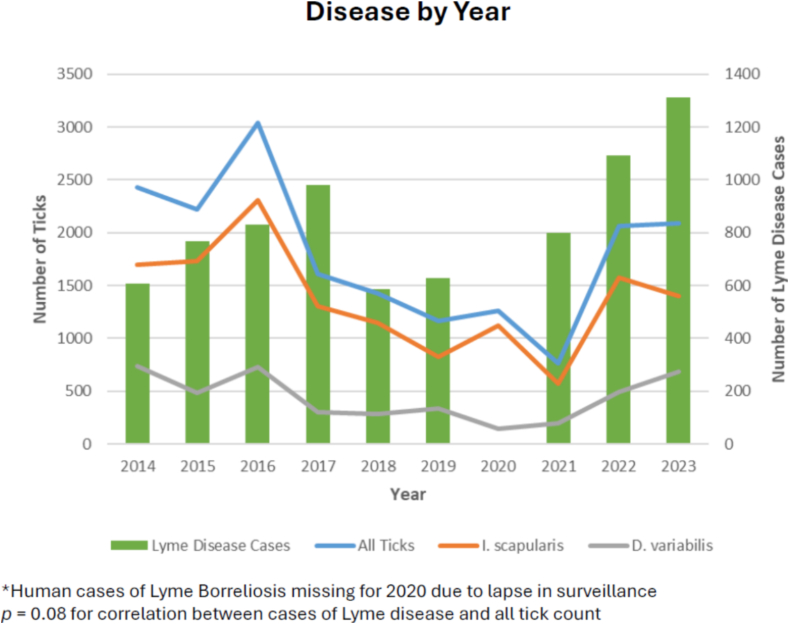
Tick Abundance Compared to Cases of Lyme Disease by Year.

There were 18,071 ticks collected by MMCD surveillance between 2014 and 2023, ranging from 767 to 3038 ticks per year, with particularly low numbers seen in 2021 in concert with a major drought. Seventy-six percent (13,680) of ticks were *I. scapularis* with the remaining (4391) being *D. variabilis* (Fig. 1). Eighty-five percent of ticks were larvae, 15% were nymphs, and none were adults. Human cases of Lyme disease ranged from 587 to 1313 with cases steadily climbing year to year since 2018. Surveillance data for Lyme disease cases was not available in 2020. A description of environmental characteristics by high and low tick abundance, categorized as above or below the 50th percentile, is provided in Supplementary Table 1. Notably, snow depth, degree days, and small mammal counts show visible differences between high and low tick abundance years. Higher numbers of human cases of Lyme disease are also observed in years with high tick abundance compared to low.

### 3.1 Predicting tick prevalence from environmental factors

Table 1 shows the estimates for the included variables in each of the nine tick prevalence outcome measures (grey rows). Employing backward selection in logistic regression, VPD was negatively associated with high tick years, in example, every one unit increase in VPD decreased the odds of a high abundance *D. variabilis* year by 0.12, or 88% (95% CI: 0.03 to 0.47). Precipitation, SWE, animal count and mouse ratio were all positively associated with the odds of having a higher tick year. These effects are most prominently seen in the mouse ratio variable where we see the odds of a high all tick density year increase by 1.58 (95% CI: 1.08 to 2.29) for every 0.1 increase in the ratio. PDSI and soil moisture showed differences in direction of association between models. AUC values were highest for the models that examined tick outcome as abundance compared to density and intensity.

**Table 1:**
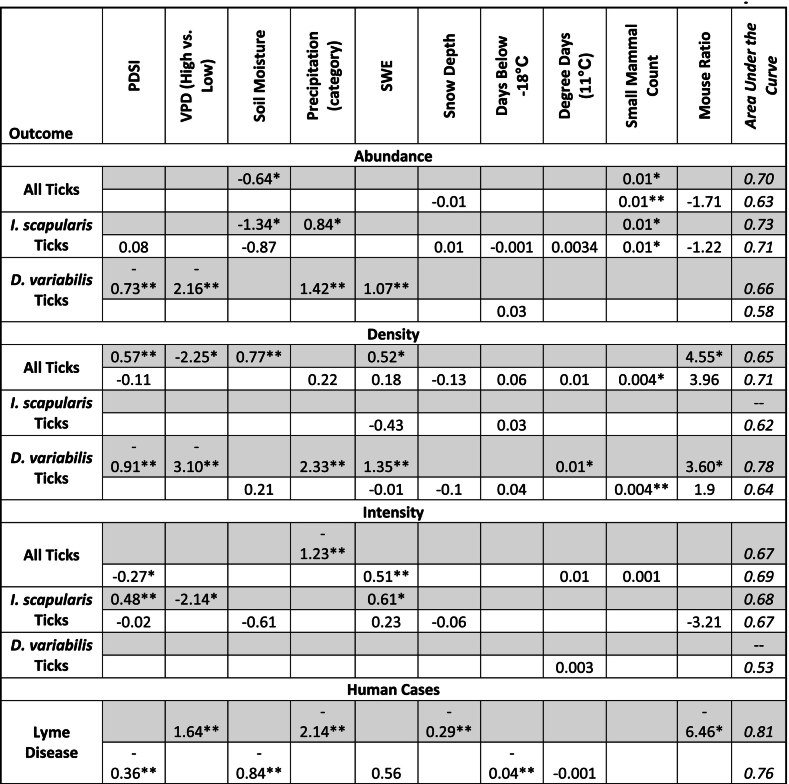
Logistic Regression Estimates for the Odds of an Upper 50 Percentile Tick Season - No Time Lag.

*Note:* In each cell of the table, regression estimates for each variable's contribution to the likelihood of having a worse season for ticks and human cases of Lyme disease are shown. The estimates are generated from one of two logistic regression models, one optimized through conventional backward selection (grey), and the other through gradient boosted machine learning (white). Empty cells indicate that those variables were excluded from the final model in the respective optimization process (grey or white). “—” in the “Area Under the Curve” column indicates that the selection process removed all variables.

*p < 0.05 **p < 0.01.

PDSI: Palmer Drought Severity Index, VPD: Vapor Pressure Deficit, SWE: Snow Water Equivalence.

Gradient boosting machine learning was performed both as an alternative exploration into environmental effects on tick prevalence and as a model building technique for logistic regression. Variables of importance and their relative influence on high or low tick years can be seen in Table 2 with darker green cells representing stronger influence on the outcome. Snow depth, small mammal count, and mouse ratio were consistently associated with tick abundance, density and intensity with relative influence ranging from 11 to 15 for snow depth, 13 to 18 for small mammal count and 13 to 16 for mouse ratio. PDSI, soil moisture and degree days were also consistently associated with tick abundance, density and intensity, although with less relative influence. SWE was associated with only density and intensity tick measures.

**Table 2:**
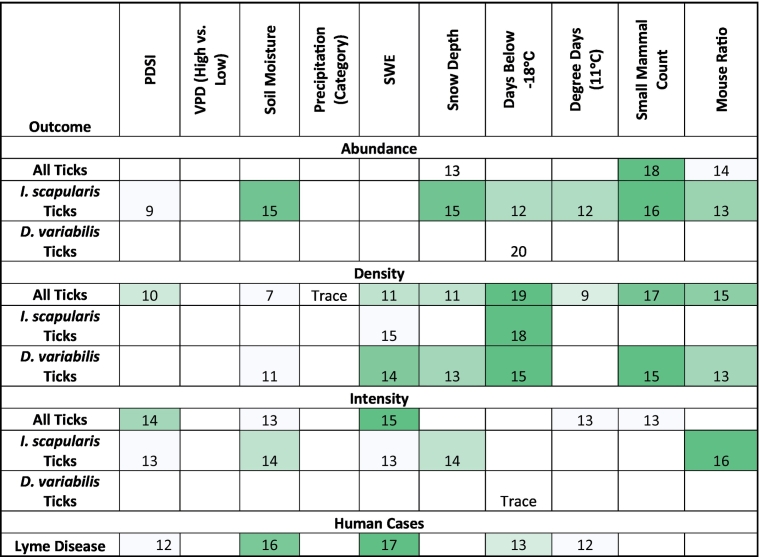
Gradient Boosting Outcomes: Variables of Importance with Relative Influence on Tick Prevalence.

*Darker green indicates higher relative influence for that outcome.

“Trace” indicates a relative influence less than 0.5.

*Empy cells indicate variable exclusion from final model.

PDSI: Palmer Drought Severity Index, VPD: Vapor Pressure Deficit, SWE: Snow Water Equivalence.

Results from integration of the gradient boosting outputs into refined logistic regression models are shown in Table 1. In general, estimates from these refined models are similar or smaller than those of the traditional backward selection approach. For example, when considering all tick density, the odds of a higher tick year in the backward selection created model for SWE was 0.52 compared to 0.18 for the gradient boosted created model. Similarly, many of the variables in the gradient boosted refined model fail to meet statistical significance. In most cases, the directionality of association for variables remains the same between the two modeling approaches, however, PDSI shows opposite directionality of estimates. These models reveal consistent signal in association with tick abundance from PDSI, soil moisture, snow depth, days below −18 °C, degree days, small mammal count, and mouse ratio.

### 3.2 Predicting lyme disease cases from environmental factors

The results of models examining predicting factors for cases of Lyme disease are provided in Table 1, Table 2. In contrast to the associations with tick abundance, logistic regression models using both backward selection and gradient boosting for variable selection show no overlap in variables included in the models. Gradient boosting shows SWE to be the most influential variable with a relative influence of 17 followed by soil moisture, cold snap days, degree days and PDSI. The backward selection built logistic regression model includes VPD, precipitation, snow depth and mouse ratio all meeting the inclusion threshold of *p* < 0.05. Nonetheless, model accuracy is similar between the two models with AUC values of 0.81 for the backward selection model and 0.76 for the gradient boosting created model.

### 3.3 Time lag effects on predicting tick prevalence and lyme disease cases from climate factors

Logistic regression outputs for models with a single year time lag can be found in Table 3. Soil moisture, SWE, snow depth, cold snap days, degree days, small mammal count and mouse ratio are consistently associated with all one-year lagged tick prevalence metrics. Increased soil moisture, SWE, and degree days in one year is protective against a high tick season one year later. Conversely, increased snow depth and cold snap days indicate increased odds of a high tick season one year later. There are examples of inconsistency with direction of association among small mammal count and mouse ratio estimates. AUC values are highest among tick abundance outcomes overall and *D. variabilis* ticks. Incorporating a one-year time lag in estimating years with high versus low Lyme disease case counts yields no results with the traditional backward selection approach, but with the gradient boosting technique, PDSI predicts Lyme cases with a high degree of accuracy. Every one unit increase in PDSI results in a 54% (95% CI: 46% to 60%) decrease in the odds of a high Lyme disease year one year later.

**Table 3:**
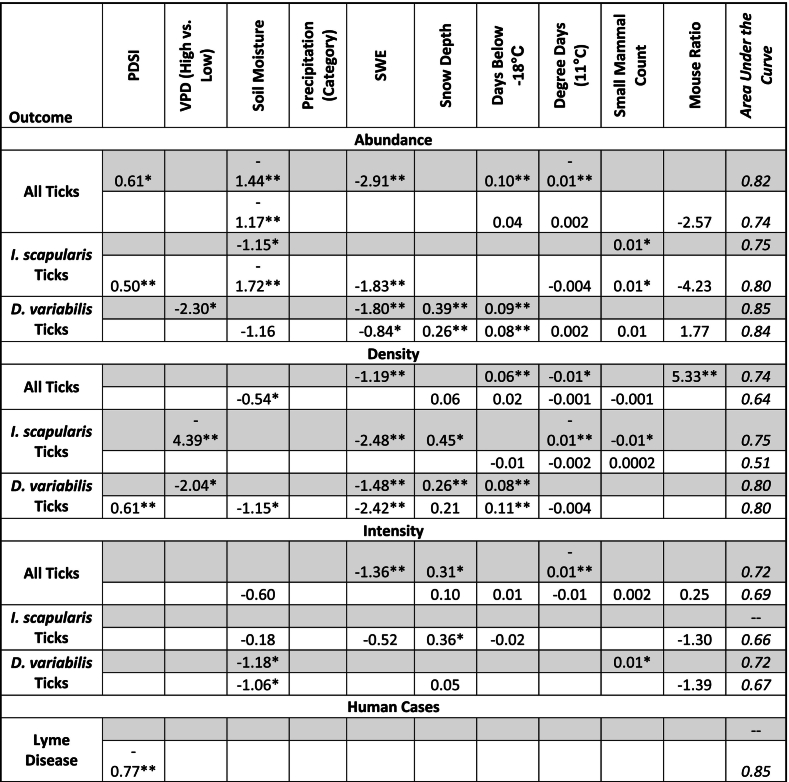
Logistic Regression Estimates for the Odds of an Upper 50 Percentile Tick Season - 1 Year Time Lag.

*Note:* In each cell of the table, regression estimates for each variable's contribution to the likelihood of having a worse season for ticks and human cases of Lyme disease are shown. The estimates are generated from one of two logistic regression models, one optimized through conventional backward selection (grey), and the other through gradient boosted machine learning (white). Empty cells indicate that those variables were excluded from the final model in the respective optimization process (grey or white). “—” in the “Area Under the Curve” column indicates that the selection process removed all variables.

*p < 0.05 **p < 0.01.

PDSI: Palmer Drought Severity Index, VPD: Vapor Pressure Deficit, SWE: Snow Water Equivalence.

Logistic regression outputs for models with a two-year lag can be found in Table 4. PDSI, soil moisture, SWE, snow depth, degree days and mouse ratio are all consistently associated with tick prevalence metrics two years later. Soil moisture and SWE are both negatively associated with high tick prevalence years, while snow depth is positively associated with tick outcomes. Mouse ratio is positively associated with tick abundance and density, but negatively with intensity measures. PDSI is negatively associated with abundance and intensity, but positively with density measures. There are inconsistencies between the modeling approaches with direction of association in cold snap days and degree days. AUC values are highest among tick abundance outcomes.

**Table 4:**
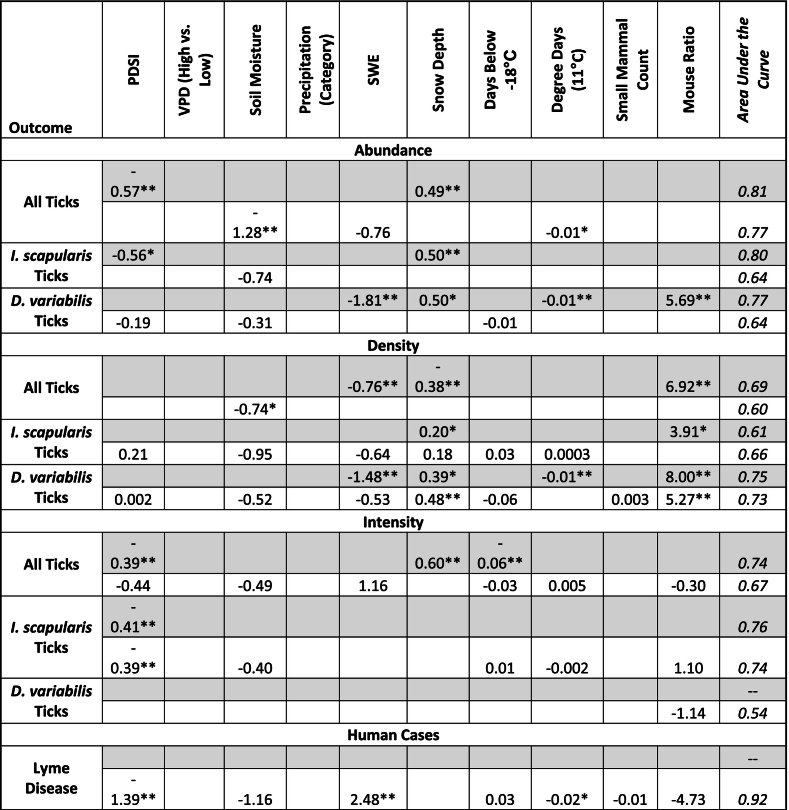
Logistic Regression Estimates for the Odds of an Upper 50 Percentile Tick Season - 2 Year Time Lag.

*Note:* In each cell of the table, regression estimates for each variable's contribution to the likelihood of having a worse season for ticks and human cases of Lyme disease are shown. The estimates are generated from one of two logistic regression models, one optimized through conventional backward selection (grey), and the other through gradient boosted machine learning (white). Empty cells indicate that those variables were excluded from the final model in the respective optimization process (grey or white). “—” in the “Area Under the Curve” column indicates that the selection process removed all variables.

*p < 0.05 **p < 0.01.

PDSI: Palmer Drought Severity Index, VPD: Vapor Pressure Deficit, SWE: Snow Water Equivalence.

Assessing Lyme disease outcomes with backward selection-built models with one- and two-year time lags yields null model selection. However, in machine learning-built models, both time lags result in high accuracy in predicting the odds of a high Lyme disease incidence year, especially with a 2-year lag. PDSI, soil moisture, degree days, small mammal count and mouse ratio are all negatively associated, and cold snap days were positively associated with the odds of a high Lyme disease year.

**Fig. 2:**
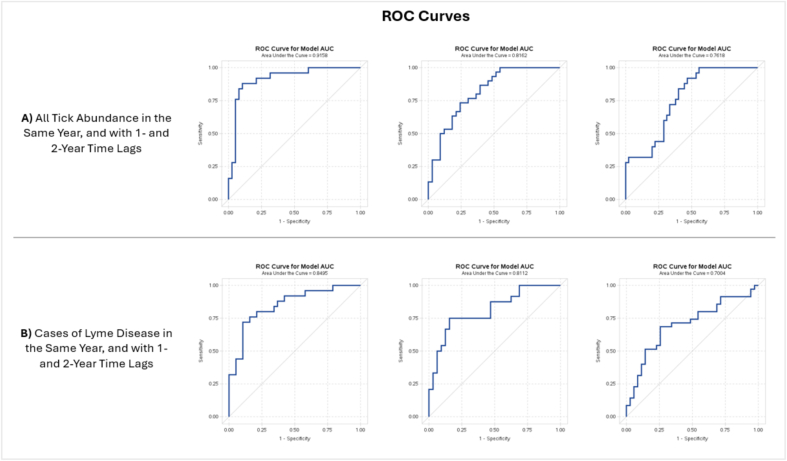
ROC Curves for (A) All Tick Abundance and (B) Cases of Lyme Disease, in the Same Year, and with 1- and 2-Year Time Lags.

A comparison of Receiver Operating Characteristic (ROC) curves across time lag effects for all tick abundance and Lyme disease cases can be found in Fig. 2. For all tick abundance measures, the AUC value is lowest with no time lag which is consistent among other tick prevalence measures. Importantly, AUC is highest with a one-year time lag and drops slightly with a two-year lag. In contrast, for Lyme disease cases, the AUC increases with each year lag, peaking at 0.92 with a two-year lag.

## 4 Discussion

Our approach integrating vector, host, environmental, and human case data together with tandem machine learning and logistic regression analyses facilitated several nuanced observations.

### 4.1 Predicting tick prevalence

Variables consistently associated with tick prevalence in conventional analysis are PDSI, VPD, precipitation and SWE. Their directionality is consistent with previous literature and suggest that higher levels of moisture lead to higher levels of ticks and more snow leads to overwinter tick survival, resulting in higher levels the following year [2], [12], [15]. In this analysis and previous literature, *D. variabilis* ticks appear to be more affected by environmental factors than *I. scapularis* ticks [16]. *I. scapularis* ticks are most affected by moisture-related variables (soil moisture, precipitation and VPD). Accuracy of the models is highest among abundance outcomes.

Distinct from traditional analyses, machine learning methods identified both environmental (cold snap days), and ecological (small mammal count and mouse ratio) variables as having the highest relative influence on tick prevalence metrics. Small mammals are the preferred source of blood meal for early life stage ticks; their count is significantly associated with *I. scapularis* abundance measures with a 46% increase in the odds of a high tick year for every additional 40 small mammals trapped. SWE and PDSI signals are consistent between the machine learning and logistic regression models.

Accuracy, as represented by AUC values for logistic regression models using gradient boosting as the selection method, largely shows lower values than that of traditional backward selection. While more conservative, when done in tandem, machine learning affords the opportunity to retain variables that may become important during seasonal variation or when propensity matching is desired.

Differences in the direction of effect of soil moisture and PDSI between the two analytic approaches may reflect the presence of an inflection point where moisture becomes detrimental to tick survival. Similarly, high SWE could imply an abundance of light fluffy snow that protects ticks in harsh winters, or a small dense layer that does not produce the same insulating effect. Future models may benefit from interaction testing between SWE and snow depth. The distribution of these variables against tick density was relatively linear though without granularity on time within the season.

### 4.2 Predicting lyme disease cases from environmental factors

Previous literature has shown that tick abundance is closely related to rates of tick-borne disease [4], [5], [17]; and we show analogous associations with human disease cases. The mouse ratio is integral to the disease ecology of Lyme disease as the white footed mouse is a highly competent reservoir of *B. burgdorferi* [18], [19], [20]. The mouse ratio reflects the degree to which the small mammal community is dominated by a single highly competent reservoir host; our findings with this metric align with the well-known ‘dilution effect’ theory [21]. In ecosystems with higher host diversity, the presence of alternative, less competent blood-meal sources reduce the availability of feeding opportunities on *P. leucopus*. Consequently, a high mouse ratio indicates a community lacking this biodiversity buffer, leading to higher pathogen transmission rates within the tick population, which ultimately drives increased rates of disease in humans. There is an intrinsic time lag effect on mouse ratio as humans are thought to most often be infected from nymphal ticks. Modeling this relationship is complicated by regional variations in tick lifespans; harsh winters and limited growing degree days in the Upper Midwest frequently extend ticks life cycle [22]. This expanded timeline complicates temporal modeling, but underscores fundamental differences in ecology between the Midwest and Northeast. Other important model variables such as PDSI, precipitation and degree days may predict tick-borne disease either through tick prevalence or meteorologic effects whether and how people experience the outdoors.

### 4.3 Time lag effects on predicting tick prevalence and lyme disease cases

Comparing models created with zero-, one-, and two-year time lags reveal the highest accuracy for tick measures in one year (Fig. 2 – Panel A). Results for PDSI, soil moisture and SWE become more consistent in directionality with time lags. Small mammal count and mouse ratio associations become less consistent with time lags, suggesting that their influence is more proximate to relevant vector, host, and human exposure events. Combining the previous year's PDSI, soil moisture and SWE with the current year's small mammal prevalence and mouse ratio may provide an early warning for high tick prevalence years indicating increased resources and vigilance in surveillance as well as risk communication to those embracing the outdoors [23].

While same and one-year delayed metrics optimize predictive accuracy, the statistical significance of the two-year environmental time lag also warrants biological consideration. Because our dataset is predominantly composed of larval *I. scapularis*, which hatch in the focal year of surveillance, this two-year delay highlights the multi-year life cycle of this vector. Environmental conditions two years prior likely influence survival, feeding success, and density of preceding cohorts. Alternatively, two-year lags may reflect broader ecological cascades, such as climate impacts on forest productivity and host availability in previous years, which may dictate the reproductive success of adult ticks and the resulting larval density in the focal year.

Machine learning built models were successful in discerning time-lag influences in ways not achieved with conventional logistic regression approaches (Fig. 2 – Panel B). Our integrated analysis approach can be replicated to fill gaps in disease prediction where traditional methods have previously failed; and useful in elucidating aspects of climate-influenced vector ecology. For instance, across all gradient boosted models (zero-, one- and two-year time lag) PDSI is negatively associated with a high Lyme disease case year with the regression estimate becoming more prominent with each successive lag year added, suggesting a two-year delay of effect.

### 4.4 Strengths and limitations

The MMCD dataset represents a rare example of continuous, multi-decade active vector surveillance intended to explore the prevalence, geographic distribution and movement over time of the *I. scapularis* tick. This surveillance has successfully targeted the early life stages of *I. scapularis* ticks which may lead to bias in the results, while also potentiating the lag-time assessments incorporated here. Cases of Lyme disease from MDH are recorded by county of residence which may not reflect locations of exposure. The case definition for reporting Lyme disease changed in 2022. For high incidence states like Minnesota, this change eliminated the use of clinical criteria like erythema migrans to classify cases. When probable and confirmed cases in years prior to 2022 are combined, total MDH case counts are comparable across the definition change [6]. This may have reduced the number of Lyme disease cases reported and diminish statistical power. The evaluation of time lag effects allows for a more accurate prediction of high tick seasons using a multi-year approach. The influence of environmental factors on tick prevalence may be subject to threshold effects that were not identified in this work. Additional years of data and expanded geographic range would be helpful in assessing time lag effects of cases of Lyme disease. Future directions may include increasing sensitivity through evaluating timing of meteorologic factors on tick prevalence measures as these factors are likely to have varying effects depending on their timing within the season.

## 5 Conclusions

Climate and other environmental factors allow effective prediction of high tick abundance and Lyme disease case burden years in Minnesota up to two years in advance, particularly when outputs of conventional and machine learning analyses are incorporated. Environmental factors predict high tick abundance best for the same and subsequent year. Adding the same year small mammal count and mouse ratio may enhance the predictive power of Palmer Drought Severity Index (PDSI), soil moisture and snow water equivalent (SWE) in the previous year, offering a robust option for early warning. In concert with real-time assessments during the same season, early detection of increased tick exposure risk presents an opportunity for public health intervention through public education on tick attachment prevention, early detection of an attached tick and proper removal. Opportunities exist for One Health surveillance and health system strengthening, and social mobilization for risk-reduction strategies, including prevention, raising awareness of signs and symptoms to monitor following discovery of an attached tick, and early warning to healthcare providers in the region with materials to familiarize them with risk factors, signs and symptoms, and best practices for Lyme disease case management.

## Availability of data and materials

TerraClimate and MNDNR data used in this study are open-access and freely available. Data obtained from MMCD and MDH were provided by request and cannot be publicly shared; the authors do not have permission to distribute these datasets.

## CRediT authorship contribution statement

**Kathleen E. Angell:** Writing – review & editing, Writing – original draft, Visualization, Methodology, Formal analysis, Conceptualization. **Janet Jarnefeld:** Writing – review & editing, Data curation. **Elizabeth K. Schiffman:** Writing – review & editing, Data curation. **M. Jana Broadhurst:** Writing – review & editing, Supervision. **Jianghu James Dong:** Writing – review & editing, Methodology. **Abraham Degarege:** Writing – review & editing, Methodology. **Roberto Cortinas:** Writing – review & editing, Supervision. **David M. Brett-Major:** Writing – review & editing, Writing – original draft, Supervision, Conceptualization.

## Ethics approval and consent to participate

This work was determined to not be human subjects research by the University of Nebraska Medical Center Institutional Review Board.

## Funding

The authors declare that no funding was obtained for this work.

## Declaration of competing interest

The authors declare that they have no known competing financial interests or personal relationships that could have appeared to influence the work reported in this paper.

## Data Availability

TerraClimate and MNDNR data are open-access and freely available. MMCD and MDH data were provided by request; the authors do not have permission to distribute these datasets.

## Appendix A Supplementary data

Supplementary **Table1: Environmental Characteristics by Tick Abundance Level**
