# Supplementary material for: Climate and other environmental factors predict tick abundance and Lyme cases in Minnesota one and two years in advance
Source: One Health. 2026 Jul 3;23:101507. doi: 10.1016/j.onehlt.2026.101507 (PMC13380151; doi:10.1016/j.onehlt.2026.101507)
Supplement: Supplementary file 1 — Supplementary Table1: Environmental Characteristics by Tick Abundance Level [file mmc1.docx]

| **Supplementary Table 1: Environmental Characteristics by Tick Abundance Level** | | | | |
| --- | --- | --- | --- | --- |
|  | **All Tick Abundance Years** | **Lower 50th percentile** | **Upper 50th percentile** | ***p-*value** |
|  | Mean ± Standard Deviation | | |  |
| **PDSI** | 2.4 ± 2.4 | 2.8 ± 2.7 | 2.1 ± 2.2 | 0.24 |
| **VPD** | 0.8 ± 0.1 | 0.8 ± 0.1 | 0.8 ± 0.1 | 0.99 |
| **Soil Moisture** | 2.4 ± 0.7 | 2.5 ± 0.8 | 2.3 ± 0.5 | 0.23 |
| **Precipitation (inches)** | 3.9 ± 0.6 | 3.9 ± 0.6 | 3.8 ± 0.5 | 0.94 |
| **SWE (inches)** | 2.6 ± 0.9 | 2.5 ± 1.0 | 2.6 ± 0.8 | 0.90 |
| **Snow Depth (inches)** | 3.2 ± 2.5 | 3.0 ± 1.6 | 3.4 ± 3.2 | 0.48 |
| **Days Below -18°C** | 29.1 ± 12.1 | 27.9 ± 9.9 | 30.3 ± 13.9 | 0.42 |
| **Degree Days (11°C)** | 303 ± 82 | 294 ± 87 | 312 ± 78 | 0.36 |
| **Small Mammal Count** | 156 ± 86 | 135 ± 71 | 178 ± 95 | 0.03 |
| **Mouse Ratio** | 0.85 ± 0.08 | 0.86 ± 0.08 | 0.84 ± 0.08 | 0.37 |
| **Cases of Lyme Disease** | 121 ± 85 | 114 ± 85 | 126 ± 86 | 0.57 |
| PDSI: Palmer Drought Severity Index, VPD: Vapor Pressure Deficit, SWE: Snow Water Equivalence | | | | |
